# Intrapocket application of tea tree oil gel in the treatment of stage 2 periodontitis

**DOI:** 10.1186/s12903-021-01588-y

**Published:** 2021-05-05

**Authors:** Maha R. Taalab, Sabah Abdelhady Mahmoud, Riham M. El Moslemany, Dania M. Abdelaziz

**Affiliations:** 1 Department of Oral Medicine, Periodontology, Oral Diagnosis and Oral Radiology, Faculty of Dentistry, Alexandria University, Champolion St. Azarita, Alexandria, 21521 Egypt; 2 Department of Medical Biochemistry and Molecular Biology Department, Faculty of Dentistry, Alexandria University, Alexandria, Egypt; 3 Department of Pharmaceutics Department, Faculty of Pharmacy, Alexandria University, Alexandria, Egypt

**Keywords:** Tea tree oil gel, Matrix metalloproteinase-8, Periodontitis, Anti-inflammatory

## Abstract

**Background:**

The gold standard in treatment of periodontitis is mechanical removing of dental biofilm but using local delivery drugs as adjunctive to SRP is widely used to modulate inflammatory host and eradicate microbes. Tea tree oil (TTO) has a broad-spectrum antimicrobial, anti-inflammatory, antifungal, antiviral, antioxidant effect. This study aimed to assess clinically and biochemically the effect of intrapocket application of TTO (*Melaleuca alternifolia*) gel adjunctive to scaling and root planing (SRP) in the treatment of stage 2 (moderate) periodontitis and to correlate the biochemical levels with clinical response.

**Methods:**

A randomized, controlled clinical trial was conducted on thirty patients with stage 2 periodontitis. Patients were equally divided into two groups: Control Group treated with (SRP) alone and Test Group treated with SRP and locally delivered 5% TTO gel. Clinical assessment included pocket probing depth (PPD), clinical attachment loss (CAL), gingival index (GI) and bleeding on probing (BOP) measured at baseline and after 3 and 6 months. The level of matrix metalloproteinase-8 (MMP-8), in the gingival crevicular fluid (GCF) was also assessed at baseline and after1, 3 and 6 months by Enzyme-linked immunosorbent assay (ELISA) kit. Chi-square, Student t- tests, Mann–Whitney U test and Spearman correlation were the statistical tests used in the study.

**Results:**

An improvement of all clinical and biochemical parameters was observed (at *p* < 0.001) in both groups. A significant difference between the two groups was found in both clinical and biochemical parameters.

**Conclusion:**

The local delivery of TTO gel adjunctive to SRP proved to be effective in the treatment of stage II periodontitis.

*Trial registration* The study was retrospectively registered at clinicaltrials.gov NCT04769271, on 24/2/2021.

## Introduction

Periodontitis is a common microbial infection-induced inflammatory disease that results in destruction of the periodontium leading to tooth loss. The onset, progression and severity of the disease depend mainly on the host immune-inflammatory response to microbial dental plaque. The immune cells activation stimulates enzymes release which results in destruction of both connective tissue and bone [1–4].

It was reported that the pathogenesis and progression of periodontitis is regulated by several inflammatory mediators such as galectin-3, prostaglandins, interleukins, metalloproteases, and upregulation of C‐reactive protein (CRP)released from fibroblast, epithelial cells, neutrophils and macrophages, into the bloodstream in reaction to periodontal bacteria and the authors proved that serum and salivary Galectin‐3 levels were considered to be a valuable prognostic early marker of periodontitis and coronary heart diseases [5].

Moreover salivary interleukin-6 (IL-6) levels in periodontitis patients were also assessed by Isola etal (2020) as an inflammatory biomarker and they found that periodontitis patients presented significant higher salivary IL-6 levels compared to healthy subjects and that salivary IL-6 levels were inversely associated with the number of teeth in those patients [6].

Periodontal tissue destruction is controlled mainly by matrix metalloproteinases (MMPs), a family of zinc and calcium-dependent proteolytic enzymes, which are secreted by cells of the immune system such as polymorphonuclear leukocytes and fibroblasts [7–9].

Metalloproteinases are classified among a large group of metal-dependent endopeptidases formed by connective tissue cells [1, 2, 7]. They are secreted as latent proenzymes and activated in the extracellular environment. Activation of MMPs occurs by breaking the bond between Zn^+2^ and cysteine, resulting in the blocking of the reactivity of the enzyme [1, 2, 8].

Matrix metalloproteinase-8 (MMP-8) (neutrophilic collagenase; collagenase-2) is the main cause for type I, II and III collagen destruction. It was reported that MMP-8 is the main enzyme in the salivary fluid and gingival tissue, and most probably responsible for the destruction of the periodontal tissues with active inflammation [7, 10–13].

Previous researches proved that there is a relation between the increased levels of MMP-8 in saliva, and GCF and clinical periodontal parameters including mean Pocket depth (PD), Gingival index (GI), and Bleeding on probing (BOP) and reported that MMP-8 level can reflect periodontal disease activity [14–16].

The main goal of the periodontal treatment is to remove dental plaque to decrease the number of bacteria and so stop inflammation and disease progression. The use of local chemotherapeutic agent in adjunctive to Scaling and root planing (SRP) is considered the first treatment of choice, due to its limited invasiveness [17, 18].

It was reported that periodontitis is classified according to the severity and complexity of the treatment into four stages stage 1: mild periodontitis, stage 2: moderate periodontitis, stage 3: severe periodontitis can lead to tooth loss, and stage 4: periodontitis in sever form can lead to loss of all dentition. While regarding disease progression and predicted treatment response periodontitis is classified into three grades including grade A, grade B and grade C [19].

Accurate diagnosis can assess the severity, activity and progression of periodontitis leading to the correct treatment plan. There are several diagnostic procedures such as probing pocket depth, clinical attachment loss, bleeding on probing, plaque index, grade of mobility and amount of alveolar bone loss by radiographs [20].

Depending on the severity of inflammation and degree of periodontal destruction, non surgical periodontal treatment in the form of SRP has been combined with systemic antibiotics with variable degrees of success [21].

Locally delivered drug results in a high concentration of this drug in the periodontal pocket, which can reach the periodontal tissues as well. This dual effect on pocket microflora provides 100‑fold higher therapeutic doses of the agent in subgingival areas than systemic antimicrobial therapy. Also results in enhancement of clinical parameters without systemic side effects [22].

Natural products have served as a main source of drugs for centuries and about half of pharmacological products in use now are derived from natural materials [23]. Tea tree oil (TTO) belongs to these materials as it is derived from the paper bark tea tree, which has an anti‑inflammatory, antioxidant, antifungal, antiviral, and broad‑spectrum antimicrobial effects [24–27]. Few studies stated the beneficial effect of the locally delivered of TTO on the management of periodontitis [28, 29].

1,8-Cineole and Terpinen-4-ol are the main active components of TTO and it was reported that 1,8-Cineole has anti-inflammatory properties [29, 30], and can penetrate human skin [31]. Other studies revealed that Terpinen-4-ol doesn’t have the same properties of 1,8-cineol as anti-inflammatory component [26, 32] only, but also has an anti-bacterial activity. The administration of TTO has the same antimicrobial property as chlorhexidine (CHX), with different action mechanisms. They have the same antibacterial, antiviral and antifungal properties [33–36].

This study was performed to provide an evidence about the clinical effect of using of TTO gel topically as an herbal product adjunctive to SRP for the treatment of moderate periodontitis and also to assess its effect on MMP-8 levels in gingival crevicular fluid (GCF) as an important inflammatory mediator that control periodontal destruction. The aim of the present study was to assess clinically through measuring: pocket probing depth (PPD), clinical attachment level (CAL), GI and BOP and biochemically through measuring the MMP-8 levels in GCF the effect of intrapocket application of TTO (Melaleuca alternifolia) gel adjunctive to SRP on the management of stage 2 (moderate) periodontitis and to correlate the GCF levels of MMP-8 with clinical response. The null hypothesis was that there was no difference in clinical or biochemical parameters between the two groups assessed.

## Methods

### Preparation of tea tree oil gel

Tea tree oil 5% gel (Sigma Aldrich® Steinheim, Germany) for local sub gingival application was prepared by the Department of Pharmaceutics, Faculty of Pharmacy.

The gelling agent; Carbopol 940 (1% w/v) was first soaked in distilled water for 2 h then TTO dissolved in an appropriate amount of propylene glycol was added to the Carbopol dispersion. Methyl paraben 0.2% w/v dissolved in preheated water was used as a preservative. The gel mixture was then magnetically stirred for 30 min. Finally, pH was adjusted using 1 N NaOH added dropwise with gentle stirring with a spatula until the desired pH value (6.5–7) was reached. The gel was sterilized by autoclaving at 110 °C for 20 min.

### Study population

A six-month, parallel randomized controlled clinical trial was conducted on thirty patients at the Department of Oral Medicine, Periodontology, Diagnosis and Oral Radiology, Faculty of Dentistry, Alexandria University between November 2019 and August 2020. Ethical approval was obtained from the Research Ethics Committee at the Faculty of Dentistry, Alexandria University, Egypt (IRBNO: 00010556- IORG:0,008839). The study was registered at clinicaltrials.gov NCT04769271, on 24/2/2021.

The objective of the study and the methods used in it were described to the patients and all of them signed an informed consent. The study was performed in accordance with the Helsinki declaration.

#### Inclusion criteria

Patients of both sexes with age range 25–50 participated in the study if diagnosed with stage 2, grade B periodontitis according to the 2017 World Workshop on the Classification of Periodontal and Peri-Implant Diseases and Conditions [19].

This diagnosis was confirmed by the presence of CAL 3–4 mm, BOP and radiographic horizontal bone loss related to the coronal third of the root (15%-33%). The patients also showed no teeth loss due to periodontitis. On assessing the severity and distribution of the disease, these patients had CAL 3–4 mm < 30% of the teeth involved with periodontitis. Patients with CAL caused by non-periodontal causes were excluded from the study.

Moreover, the grade of periodontitis was assessed by radiographic bone loss/age % which was 0.25–1% [37]. Radiographic bone loss was assessed from the tooth showing the most bone destruction. Only patients having CAL 3–4 mm and BOP in proximal tooth surface and who could maintain an O'Leary plaque index ≤ 10% proceeded into the study [38].

#### Exclusion criteria

Patients were excluded if they had any systemic disease that may affect the treatment outcomes, or if the patients were smokers, pregnant, or receiving contraindicated medications, chemotherapy, or radiotherapy in the previous year [10]. Patients were screened against these criteria during the study period and recruited if they were eligible.

### Sample size estimation

Sample size was estimated assuming alpha error = 5% and study power = 80%. Mean ± SD pentraxin levels were = 0.12 ± 0.03 when tea tree oil was used after SRP, and = 0.35 ± 0.24 when tea tree oil was not used [23]. MMP-8 is a periodontal inflammatory mediator, and is assumed to be more predictive in diagnosing periodontitis than pentraxin [39]. Based on comparison of means, sample size was calculated [31] to be 10 per group, increased to 11 to compensate for loss during follow up. The minimum total sample size = 22.

### Grouping and randomization

Thirty patients with stage 2 periodontitis were equally divided into 2 groups: Group I (control group, mean age28.9 ± 6.3 years) was treated with SRP only and Group II (test group, mean age 30.5 ± 5.6 years) was treated with SRP combined with intrapocket application of TTO gel. Patients complying with the inclusion criteria were randomly assigned using a computer-generated list of random numbers to one of the two groups [40] in blocks of four and the allocation sequence was handed to an assistant not directly involved with the study. Patients were sequentially numbered in a list and the number of each patient was written on an opaque envelope that included identical pieces of paper that were folded after adding the group to which the patient was assigned. On the day of the intervention, the assistant retrieved the envelope carrying the patient number, opened it and the intervention assigned to the patient was provided.

### Treatment

After completion of baseline measurements for both clinical and biochemical evaluation, the two groups received full mouth SRP using hand instruments and ultrasonic scalers and oral hygiene instructions.

**Fig. 1:**
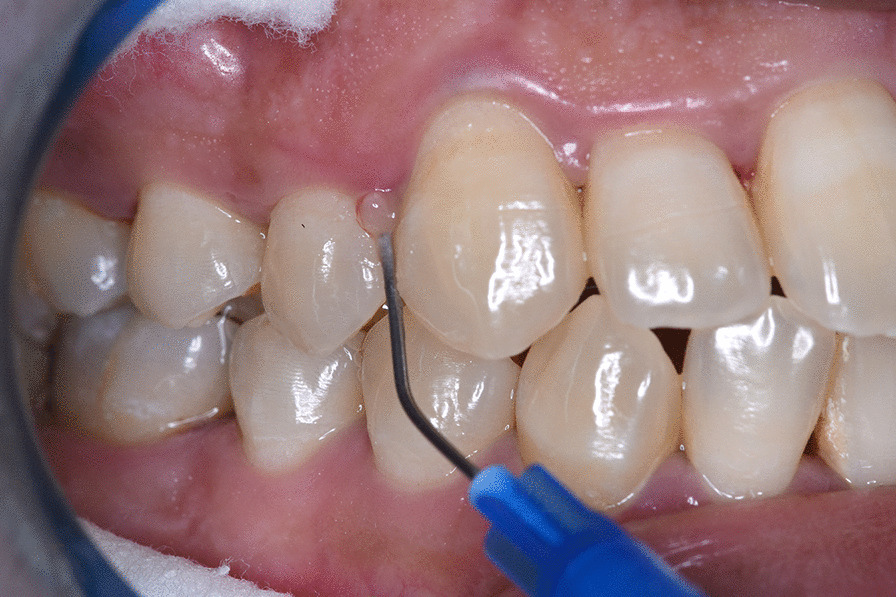
Intrapocket application of Tea Tree oil gel in test group

For test group only, partial isolation was performed for the tested site followed by drying with air. A bent, blunt-end needle syringe was used to inject the TTO gel in the pocket assuring that its tip reaches the deepest point in the pocket. As the gel appeared at the gingival margin the needle was removed slowly from the pocket (as shown in Fig. 1). After application of approximately 0.5 mL of the gel, excess gel was removed by a sterile gauze in order to avoid any spillover effect or possible systemic effect by swallowing the gel.

Patients were instructed to follow strict oral hygiene measures during the study period. They were also asked not to use the toothbrush at selected site after TTO gel application for 24 h and not to chew hard or sticky foods at the gel placement sites. On the subsequent recall visit, the clinical parameters and any adverse effects were recorded.

### Clinical assessment

Periodontal clinical parameters were recorded at 3 and 6 months after treatment, including: PPD, CAL, GI [41] and BOP [42] which was assessed within 15 s after probing, using a dichotomous scoring system (**+ **and −) for presence or absence, respectively). All measurements were recorded at tested sites with graduated William's probe by a single blinded pre-calibrated clinician who carried out double assessments for the clinical examination. Intraexaminer reliability was for probing depth 0.380 per test group and 0.250 per control group. For CAL was 0.392 per test group and − 0.142 per control group as personal coefficient test.

### Biochemical assessment

**Fig. 2:**
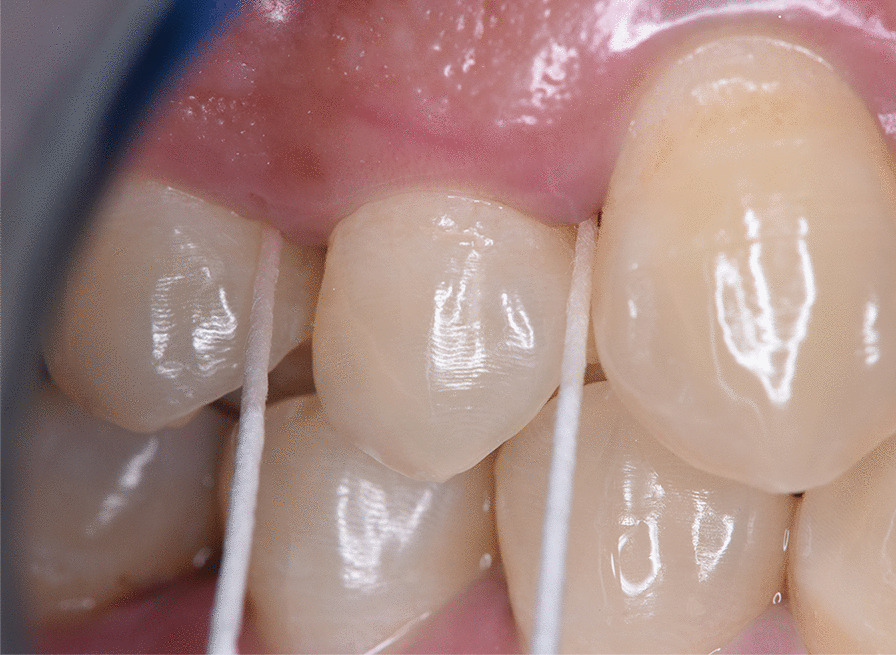
Collection of GCF sample using a prefabricated paper point for the assessment of MMP-8 level

In all patients, GCF sample was taken from the area showing the deepest pocket depth around the area received the treatment, from study and control sides. The samples were collected at baseline, and after 1, 3 and 6 months following treatment. For each site GCF sample was collected by using prefabricated paper points [43], (as shown in Fig. 2) which were inserted into the pockets after drying and complete isolation of saliva using cotton rolls and high suction unit, until resistance is felt, and kept there for 30 s [43]. Any paper point with blood contamination was discarded. The samples were diluted in phosphate buffer saline (PBS) up to 1 ml [44]. After waiting for 15 min, the paper points were removed and the samples were frozen at − 20 °C for analysis of MMP-8 [44].

Human Matrix metalloproteinase 8/Neutrophil collagenase (MMP-8) ELISA Kit (Biovision Company., Ltd, Shanghai, China) was used and analysis was done according to the manufacturer instructions.

The method used was Sandwich-ELISA technique. The Microelisa stripplate present in this kit had been pre-coated with an antibody specific to MMP-8. Samples were added to the appropriate Microelisa stripplate wells and combined to the specific antibody. Then a Horseradish peroxidase (HRP)-conjugated antibody specific for MMP-8 was added to each Microelisa stripplate well and incubated, which was followed by washing away of the free components. Then Tetramethylbenzidine (TMB) substrate solution was added to each well. Only those wells that contain MMP-8 and HRP conjugated MMP-8 antibody appeared blue in color and then turn yellow after the addition of the stop solution. The optical density (OD) was measured spectrophotometrically at a wavelength of 450 nm. The OD value was proportional to the concentration of MMP-8. The concentration of MMP-8 in the samples was calculated using a standard curve.

### Statistical analysis

Data were sent to the computer and analyzed using IBM SPSS software package version 20.0. (Armonk, NY: IBM Corp). The patient was the unit of analysis for both clinical and biochemical parameters at baseline and follow-up assessments. Qualitative data were in percent number. The Kolmogorov–Smirnov test was used to measure the distribution and normality. Quantitative data were in range (minimum and maximum), mean, standard deviation, median and interquartile range (IQR). Comparison of gender and age between the two study groups were done using chi-square and Student t- tests, respectively. Comparisons of periodontal parameters and MMP-8 level between the two study groups were done using Mann–Whitney U test. Spearman correlation was performed to assess the relation between different periodontal parameters and MMP-8 level. Significance of the obtained results was judged at the 5% level.

## Results

Out of 40 potentially eligible patients screened for the study, 30 patients (10 males and 20 females) were found eligible and included in this study with baseline evaluations and randomized into study groups (Table 1). All participants completed the six-month clinical trial. None of the patients in test group reported adverse reactions of the TTO gel. The subjective feedback of patients in group 2 on the TTO gel included cessation of bleeding upon tooth brushing and obvious relief of inflammation shortly after treatment initiation but they only complained of unpleasant taste.

**Table 1:** Comparison between the two studied groups according to demographic data

	Tests (n = 15)	Control (n = 15)	Test of Sig	p
Gender				
Male	5 (33.3%)	5 (33.3%)	χ^2^ = 0.000	1.000
Female	10 (66.7%)	10 (66.7%)		
Age (years)				
Mean ± SD	30.5 ± 5.6	28.9 ± 6.3	t = 0.705	0.486
Median (Min.–Max.)	28 (21–40)	30 (21–39)		

χ^2^: Chi square test, t: Student t-test

p: p value for comparing between the two groups

### Clinical periodontal outcome

The clinical efficacy of the TTO gel applied subgingivally for the treatment of stage 2 (moderate) periodontitis was assessed using clinical parameters at baseline and at 3 and 6 months. Statistical analysis of data is summarized in Tables 2 and 3.

**Table 2:** Comparison between the two studied groups according to different parameters

	Tests (n = 15)	Control (n = 15)	U	p
Pockets depth (mm)				
Before				
Mean ± SD	5.4 ± 1.1	5.5 ± 1.1	105.5	0.775
Median (Min.–Max.)	6^a^ (3–6)	6^a^ (3–6)		
3 months				
Mean ± SD	3.3 ± 0.6	4.3 ± 1.5	76.50	0.137
Median (Min.–Max.)	3^b^ (2–4)	4^a^ (2–6)		
6 months				
Mean ± SD	1.9 ± 0.9	2.6 ± 0.6	65.0	0.050
Median (Min.–Max.)	2^c^ (1–3)	3^b^ (1–3)		
p_0_	< 0.001*	< 0.001*		
%decrease after 3 m	34.9 ± 26.4	21.3 ± 25.5	80.0	0.187
Decrease after 6 m	3.5 ± 1.4	2.9 ± 1	80.50	0.187
%decrease after 6 m	62 ± 22	51.6 ± 16.8	69.50	0.074
Clinical attachment level (mm)				
Before				
Mean ± SD	3.3 ± 0.5	3.4 ± 0.5	97.50	0.539
Median (Min.–Max.)	3^a^ (3–4)	3^a^ (3–4)		
3 months				
Mean ± SD	1.4 ± 0.5	1.7 ± 0.6	79.50	0.174
Median (Min.–Max.)	1^b^ (1–2)	2^b^ (1–3)		
6 months				
Mean ± SD	0.4 ± 0.5	1.2 ± 0.7	45.0*	0.004*
Median (Min.–Max.)	0^c^ (0–1)	1^b^ (0–2)		
p_0_	< 0.001*	< 0.001*		
%decrease after 3 m	57.8 ± 12.4	48.3 ± 19.5	81.0	0.202
Decrease after 6 m	2.9 ± 0.6	2.2 ± 0.8	57.50*	0.021*
%decrease after 6 m	87.8 ± 15.7	64.4 ± 19.5	43.0*	0.003*

Medians of periods in the same column with common small letters are not significant

U: Mann Whitney test

p: p value for comparing between the two groups

p_0_: p value for Friedman test for comparing between the different studied periods

*: Statistically significant at *p* ≤ 0.05

**Table 3:** Comparison between the two studied groups according to different parameters

	Tests (n = 15)	Control (n = 15)	U	p
Gingival index				
Before				
Mean ± SD	1.17 ± 0.48	1.07 ± 0.36	93.0	0.436
Median (Min.–Max.)	1.25^a^ (0.25–1.75)	1.0^a^ (0.75–2.0)		
3 months				
Mean ± SD	0.27 ± 0.26	0.45 ± 0.40	84.50	0.250
Median (Min.–Max.)	0.25^b^ (0.0–0.75)	0.25^b^ (0.0–1.50)		
6 months				
Mean ± SD	0.03 ± 0.09	0.27 ± 0.20	40.0*	0.002*
Median (Min.–Max.)	0.0^b^ (0.0–0.25)	0.25^b^ (0.0–0.50)		
p_0_	< 0.001*	< 0.001*		
%Decrease after 3 m	82.4 ± 16.4	60.3 ± 26.4	60.0*	0.029*
Decrease after 6 m	1.1 ± 0.5	0.8 ± 0.3	62.0*	0.037*
%Decrease after 6 m	97.9 ± 5.5	74.7 ± 19.7	34.0*	0.001*
Bleeding on Probing %				
Before				
Mean ± SD	81.7 ± 22.1	85 ± 12.7	111.0	0.967
Median (Min.–Max.)	75^a^ (25–100)	75^a^ (75–100)		
3 months				
Mean ± SD	26.7 ± 25.8	41.7 ± 32.3	84.50	0.250
Median (Min.–Max.)	25^b^ (0–75)	25^b^ (0–100)		
6 months				
Mean ± SD	3.3 ± 8.8	26.7 ± 20	40.0*	0.002*
Median (Min.–Max.)	0^b^ (0–25)	25^b^ (0–50)		
p_0_	< 0.001*	< 0.001*		
%Decrease after 3 m	71.7 ± 27.2	52.2 ± 34.4	77.50	0.148
Decrease after 6 m	78.3 ± 20.8	58.3 ± 20.4	54.0*	0.015*
%Decrease after 6 m	96.7 ± 8.8	68.9 ± 23.2	36.0*	0.001*
Level of mmp8 (n/ml)				
Before				
Mean ± SD	2.6 ± 1.5	2 ± 1.6	91.0	0.389
Median (Min.–Max.)	2.3^a^ (0.4–6.4)	1.5^a^ (0.4–6.2)		
1 months				
Mean ± SD	1.7 ± 0.8	1.9 ± 2.4	91.0	0.389
Median (Min.–Max.)	1.8^a^ (0.4–2.8)	0.9^b^ (0.3–9.7)		
3 months				
Mean ± SD	0.6 ± 0.4	1 ± 0.7	83.50	0.233
Median (Min.–Max.)	0.5^b^ (0.2–1.9)	0.7^b^ (0.3–2.8)		
6 months				
Mean ± SD	0.3 ± 0.2	0.8 ± 0.6	46.0*	0.005*
Median (Min.–Max.)	0.2^c^ (0.2–1.1)	0.5^c^ (0.2–2.6)		
p_0_	< 0.001*	< 0.001*		
%Decrease after 3 m	69.2 ± 23.4	44.3 ± 21.1	42.0*	0.003*
Decrease after 6 m	2.3 ± 1.6	1.2 ± 1.1	62.0*	0.037*
%Decrease after 6 m	82.8 ± 16.2	56.8 ± 19	27.0*	< 0.001*

Medians of periods in the same column with common small letters are not significant

U: Mann Whitney test

p: p value for comparing between the two groups

p_0_: p value for Friedman test for comparing between the different studied periods

*: Statistically significant at *p* ≤ 0.05

All the clinical parameters, including pocket probing depth, clinical attachment level, gingival index and bleeding on probing showed statistically significant improvement from baseline through all follow up periods in the two groups (as shown in Tables 2 and 3).

Regarding PPD no significant difference was noted between the two groups at baseline, after 3 and 6 follow up periods at (*p* = 0.775, *p* = 0.137, and *p* = 0.050 respectively). No significant difference was found between mean % reduction in PPD after six months in test group when compared to control group at (*p* = 0.074).

As for CAL no statistically significant difference was noted between the two groups at baseline and after 3 months at (*p* = 0.539, *p* = 0.174 respectively) however at 6 months a significant difference in mean CAL was found between the two groups at (*p* = 0.004). Also a statistically significant difference in mean % reduction in CAL was found between the two groups after six months in favor to test group at (*p* = 0.003).

Regarding GI no statistically significant difference was noted between the two groups at baseline at (*p* = 0.318), however at 3 and 6 months there was statistically significant difference between mean GI in test group when compared to control group at (*p* = 0.005). Also a statistically significant difference was found between mean % reduction in GI after 3 and 6 months in test group when compared to control group at (*p* = 0.029 and *p* = 0.001 respectively).

Concerning BOP no statistically significant difference was noted between the two groups at baseline and at 3 months at (*p* = 0.967 and *p* = 0.250 respectively) while at 6 months there was a statistically significant difference between mean BOP in test group when compared to control group at (*p* = 0.002). Also a statistically significant difference was found between mean % reduction in BOP after 6 months in test group when compared to control group at (*p* = 0.001).

### Biochemical outcome

Gingival crevicular fluid MMP-8 values showed statistically significant reduction from baseline through all follow up periods in both test and control groups as shown in Table 1. On comparing the levels of GCF MMP-8 in the two studied groups at baseline and all follow up periods, it was found that there was a reduction in MMP-8 levels in the GCF after the application of TTO but didn't reach statistical significance at 1 and 3 months at (*p* = 0.389, *p* = 0.233 respectively), however at 6 months a significant decrease in levels of MMP-8 in GCF was found between the TTO group compared with the control group (*p* = 0.005) (as shown in Table 3).

Moreover, there was also statistically significant difference between mean % reduction in GCF MMP-8 levels after 3 and 6 months in relation to test group when compared to control group at (*p* = 0.003 and *p* < 0.001 respectively).

### Correlation between level mmp8 vs. clinical parameters

A significant positive correlation was found between the decrease of GCF MMP8 levels and clinical findings in test group however in control group no significant correlation was found between values of GCF MMP8 and clinical periodontal findings (as shown in Table 4).

**Table 4:** Correlation between level mmp8 and different parameters

Level mmp8 (n/ml) vs	Tests (n = 15)		Control (n = 15)	
	r_s_	p	r_s_	p
Pockets depth				
Before	0.425	0.115	0.271	0.328
3 months	0.520	0.047*	0.323	0.240
6 months	0.688	0.005*	0.039	0.891
Clinical attachment level (mm)				
Before	-0.349	0.202	0.441	0.100
3 months	0.535	0.040*	-0.124	0.660
6 months	0.693	0.004*	-0.083	0.768
Gingival index				
Before	0.062	0.827	-0.256	0.357
3 months	0.825	< 0.001*	0.060	0.831
6 months	0.590	0.021*	0.217	0.438
Bleeding on probing %				
Before	0.135	0.632	-0.220	0.430
3 months	0.825	< 0.001*	0.074	0.794
6 months	0.590	0.021*	0.217	0.438

r_s_: Spearman coefficient

*: Statistically significant at *p* ≤ 0.05

A coefficient correlation matrix was done to determine whether any relationships existed between clinical periodontal parameters and MMP-8 values in GCF. A summary of these results is presented in Table 3. The mean of PPD and CAL at both 3 and 6 months follow up periods were positive correlated significantly with MMP-8 in test group only at (*p* = 0.047 and *p* = 0.005 respectively) for PPD and at (*p* = 0.040 and *p* = 0.004 respectively) for CAL.

Moreover MMP-8 was also significantly positive correlated with both GI and BOP at both 3 and 6 months follow up periods at (*p* < 0.001 and *p* = 0.021 respectively) in test group for both parameters.

However, in the control group no significant correlation was found between values of MMP-8 in GCF and all clinical parameters as shown in Table 4.

So, significant positive correlations were noted only between MMP-8 values and PPD, CAL, GI and BOP of tested sites in test group.

## Discussion

Periodontitis is a chronic inflammatory disease that, if not treated, leads to loss of both soft- and hard tissue. The periodontal anaerobic pathogens are considered the stimulating agents for host response through triggering the release of proinflammatory mediators such as growth factors, cytokines, and MMPs specially MMP-8 [45]. The progression of periodontitis is usually controlled by the degree of inflammation which can be measured by inflammatory biomarkers, such as MMP-8 which is worthy investigated as both diagnostic aid and therapeutic target in periodontitis.

The rational for the use of a herbal product for the local treatment of periodontitis in the present study stemmed from their extensive natural activities, advanced safety margin, and inferior costs compared to conventional drugs. TTO was selected as it possesses antimicrobial [33], anti-inflammatory [26, 27], and antioxidant [25] properties, making it a suitable candidate for the treatment of moderate periodontitis.

In the present study, TTO gel was locally applied intra pocket and showed to be an active agent in the treatment of stage 2 (moderate) periodontitis. During the study period, the patients did not suffer from any complications following treatment. The only complaint was the unpleasant taste of the TTO gel.

The superior efficacy of test group over control group could be explained by the antimicrobial [33], anti-inflammatory [26, 27], and antioxidant [25] properties of TTO, thus, leading to reduction of both inflammatory mediators and periodontal pathogens which in turn decrease the stimulation of inflammatory cytokines giving chance for healing of periodontal tissue as observed by superior healing and clinical outcomes in the test group. MMP-8 is responsible for the beginning of collagen degradation, hence, measuring GCF MMP-8 levels can offer a site-specific diagnosis of periodontal disease, longitudinal monitoring of periodontitis and assessment of treatment outcomes [44, 46]. An increased GCF MMP-8 levels, reflects periodontal inflammation especially in clinically active phases [14, 44]. A finding of supreme importance was the substantial reduction in MMP-8 levels up to 6 months follow up period, initially found to be in very high levels, following the locally delivered of TTO gel adjunct to SRP. The reduced level of MMP-8 observed indicated clinical improvement of the periodontal disease as reflected by PPD, CAL, GI and BOP assessment.

An explanation for the improvement of both clinical and biochemical parameters is the ability of TTO components to suppress the production of TNF α, IL-1beta, IL-8, IL-10 and PGE2 which was proved through an in vitro study performed by Hart et al. [32] The authors concluded that the water-soluble components of TTO can suppress pro-inflammatory mediator production by lipopolysaccharide activated human monocytes which proves its anti-inflammatory effect. The previous cytokines are responsible for stimulation of gingival fibroblasts, to produce collagenolytic MMPs including MMP-8, therefore reduction of inflammatory cytokines by TTO could be the main cause of GCF levels of MMP-8 significant reduction in test group.

As periodontitis is a polymicrobial inflammatory disease, TTO gel efficacy could be explained not only by its anti-inflammatory effect but also its antibacterial action. Biofilm elimination in periodontitis by non-surgical SRP was performed either alone (control group) or in adjunct with TTO gel. Although SRP has caused a decrease in pathogens, it has some limitations as difficulty in reaching inaccessible areas including very deep periodontal pockets, root concavities, bifurcations, and large invaginations which can’t be treated by SRP alone specially when surgical treatment is contraindicated. Hence, TTO as an adjunct therapy was evaluated in the current study. It is well agreed that in the presence of biofilms, efficacy of topical chemotherapeutic agents against periodontal pathogens is hindered due to inadequate penetration through the biofilm. A previous study [31] reported the lipophilic nature of TTO and the penetration enhancing effect of its components which facilitate its diffusion through the epithelium making its anti-inflammatory and antibacterial properties more potent. Moreover, the antibiofilm action of TTO is well reported.

The results obtained in the current study were consistent with those obtained by Elgendy et al. [23] assessing efficacy of TTO on chronic periodontitis. The authors proved that TTO gel significantly reduced inflammation and bleeding of the gingiva in people with chronic periodontitis. Similarly, Ripari et al. [47] reported enhanced reduction in PD and clinical signs of inflammation by TTO compared to chlorhexidine when both were used as mouth washes for the treatment of gingivitis. This is in agreement with the results obtained by Salvatori et al. [48] evaluating the antibacterial and anti-inflammatory efficacy of a TTO mouth rinse compared to chlorhexidine 0.12%, essential oils, and a placebo. They concluded that the TTO mouthwash is an effective safe adjunct in the treatment of gingivitis as it resulted in improvement of bleeding index, plaque index and gingival index owing to its anti-inflammatory properties. Also, a two times daily application of TTO as a toothpaste resulted in significant decrease in the gingival inflammation and bleeding in people with severe gingivitis, proving the anti-inflammatory effect of TTO [28].

To our knowledge, this is the first study in literature that monitor the effect of topical application of TTO gel on GCF MMP-8 levels and proved that it resulted in a significant decrease which can also explain the statistically significant difference between mean % reduction in all clinical findings after six months in test group when compared to control group. The present study is limited by the length of the follow-up period, the number of patients included and the focus on one type of periodontitis only. Future studies including more participants and longer follow-up during maintenance phase should confirm these findings and give us better insight into the role of TTO in the treatment of different stages and grades of periodontitis.

## Conclusions

A proof of concept was provided for the clinical benefit of TTO gel as a novel adjunctive to SRP in the treatment of stage 2 (moderate) periodontitis using a randomized controlled clinical trial on thirty patients. The results of the current study proved that a single application of locally delivered TTO gel provides better therapeutic effects than SRP alone. Also, it proved the efficacy of TTO as an anti‑inflammatory agent as it results in significant reduction in levels of MMP-8 in GCF which correlated positively with reductions in clinical parameters related to disease severity. Thus, using intrapocket TTO gel as an adjunct to SRP was a safe and effective treatment that can improve both clinical and biochemical findings up to six months period.

## Data Availability

The datasets used and/or analysed during the current study available from the corresponding author on reasonable request.

## Abbreviations

TTO: Tea tree oil
SRT: Scaling and root planning
PPD: Pocket probing depth
CAL: Clinical attachment loss
GI: Gingival index
BOP: Bleeding on probing
GCF: Gingival crevicular fluid
ELISA: Enzyme-linked immunosorbent assay
CRP: C-reactive protein
IL-6: Interleukin-6
MMPs: Matrix metalloproteinases
MMP-8: Matrix metalloproteinase-8
PD: Pocket depth
CHX: Chlorhexidine
PBS: Phosphate buffer saline
HRP: Horseradish peroxide
TMB: Tetramethylbenzidine
OD: Optical density
IQR: Interquartile range
